# *Leishmania* Encodes a Bacterium-like 2,4-Dienoyl-Coenzyme A Reductase That Is Required for Fatty Acid β-Oxidation and Intracellular Parasite Survival

**DOI:** 10.1128/mBio.01057-20

**Published:** 2020-06-02

**Authors:** Geo Semini, Daniel Paape, Martin Blume, M. Fleur Sernee, Diego Peres-Alonso, Sébastien Calvignac-Spencer, Jörg Döllinger, Stefan Jehle, Eleanor Saunders, Malcolm J. McConville, Toni Aebischer

**Affiliations:** a Mycotic and Parasitic Agents and Mycobacteria (FG16), Department of Infectious Diseases, Robert Koch Institute, Berlin, Germany; b Institute of Immunology and Infection Research, The University of Edinburgh, Edinburgh, United Kingdom; c Wellcome Centre for Integrative Parasitology, University of Glasgow, Glasgow, United Kingdom; d Metabolism of Microbial Pathogens (NG2), Robert Koch Institute, Berlin, Germany; e Department of Biochemistry and Molecular Biology, Bio21 Molecular Science and Biotechnology Institute, University of Melbourne, Parkville, Victoria, Australia; f Departamento de Parasitologia, Instituto de Biociências, Universidade Estadual Paulista, Botucatu, SP, Brazil; g Epidemiology of Highly Pathogenic Microorganisms, Robert Koch Institute, Berlin, Germany; h Proteomics and Spectroscopy (ZBS 6), Robert Koch Institute, Berlin, Germany; i Department of NMR-supported Structural Biology, Leibniz-Institute for Molecular Pharmacology, Berlin, Germany; Harvard T. H. Chan School of Public Health

**Keywords:** Kinetoplastida, lateral gene transfer, mitochondrial metabolism, virulence factors

## Abstract

*Leishmania* spp. are protozoan parasites that cause a spectrum of important diseases in humans. These parasites develop as extracellular promastigotes in the digestive tract of their insect vectors and as obligate intracellular amastigotes that infect macrophages and other phagocytic cells in their vertebrate hosts. Promastigote-to-amastigote differentiation is associated with marked changes in metabolism, including the upregulation of enzymes involved in fatty acid β-oxidation, which may reflect adaptation to the intracellular niche. Here, we have investigated the function of one of these enzymes, a putative 2,4-dienoyl-coenzyme A (CoA) reductase (DECR), which is specifically required for the β-oxidation of polyunsaturated fatty acids. The *Leishmania* DECR shows close homology to bacterial DECR proteins, suggesting that it was acquired by lateral gene transfer. It is present in other trypanosomatids that have obligate intracellular stages (i.e., Trypanosoma cruzi and *Angomonas*) but is absent from dixenous parasites with an exclusively extracellular lifestyle (i.e., Trypanosoma brucei). A DECR-green fluorescent protein (GFP) fusion protein was localized to the mitochondrion in both promastigote and amastigote stages, and the levels of expression increased in the latter stages. A Leishmania major Δ*decr* null mutant was unable to catabolize unsaturated fatty acids and accumulated the intermediate 2,4-decadienoyl-CoA, confirming DECR’s role in β-oxidation. Strikingly, the L. major Δ*decr* mutant was unable to survive in macrophages and was avirulent in BALB/c mice. These findings suggest that β-oxidation of polyunsaturated fatty acids is essential for intracellular parasite survival and that the bacterial origin of key enzymes in this pathway could be exploited in developing new therapies.

## Abstract (precis)

The Trypanosomatidae are protozoan parasites that infect insects, plants, and animals and have evolved complex monoxenous (single host) and dixenous (two hosts) lifestyles. A number of species of Trypanosomatidae, including *Leishmania* spp., have evolved the capacity to survive within intracellular niches in vertebrate hosts. The adaptations, metabolic and other, that are associated with development of intracellular lifestyles remain poorly defined. We show that genomes of *Leishmania* and Trypanosomatidae that can survive intracellularly encode a 2,4-dienoyl-CoA reductase that is involved in catabolism of a subclass of fatty acids. The trypanosomatid enzyme shows closest similarity to the corresponding bacterial enzymes and is located in the mitochondrion and essential for intracellular growth of *Leishmania*. The findings suggest that acquisition of this gene by lateral gene transfer from bacteria by ancestral monoxenous Trypanosomatidae likely contributed to the development of a dixenous lifestyle of these parasites.

## INTRODUCTION

*Leishmania* spp. are flagellated protozoan parasites (order Trypanosomatida, phylum Kinetoplastida) that cause a spectrum of diseases, ranging from localized cutaneous ulcers to disseminating, lethal visceral leishmaniases (1). *Leishmania* spp. have a dixenous lifestyle, infecting both insect and vertebrate hosts. Flagellated extracellular promastigotes reside in the digestive tract of their sandfly vector and are transmitted into the skin of the mammalian host during a blood meal. After uptake by host phagocytes, promastigotes differentiate to nonflagellated intracellular amastigotes and replicate within the phagolysosomal compartment of macrophages, known as the parasitophorous vacuole (PV) (2).

Although the taxonomy and phylogeny of Trypanosomatidae are still under investigation (3–5), it is now accepted that all known trypanosomatids are parasitic and found primarily in insects (6). In contrast to monoxenous trypanosomatids, which exclusively infect and reside within a single invertebrate host, dixenous *Leishmania* and *Trypanosoma* species alternate between insect and vertebrate hosts and have been extensively investigated due to their medical relevance. The dixenous and intracellular lifestyle is likely to have been associated with gene acquisitions and losses that arose from lateral gene transfer (LGT) from bacteria (7–9). Indeed, recent bioinformatic analyses suggested that LGT might be a common genetic process during the evolution of parasitic eukaryotes (10). Of note, monoxenous species of the genera *Angomonas* and *Strigomonas* are characterized by the presence of obligate endosymbiotic *Betaproteobacteria* that divide synchronously with their host cells (11) and that contribute to the metabolism of the parasite by providing essential nutrients such as heme and amino acids (7, 12, 13). Phylogenomic analyses have also indicated that many protein-coding genes in Leishmania major were acquired through LGT, with the main donors being *Proteobacteria* and other members of the gut microbiome of insect vectors (10, 14). Finally, LGT in *Leishmania* and related genera has been shown to be a dynamic process, with LGTs uniquely detected in *Leishmania* and being lost in other genera at a higher rate than that seen with universal trypanosomatid LGTs (15).

Infection of both insect and mammalian host implies metabolic adaptations dependent on the environment encountered by promastigotes and amastigotes. In the digestive tract of the sandfly vector, the energy and central carbon metabolism of promastigote stages likely evolved to depend on the catabolism of plant sap-derived sugars (16, 17) present in aphid honeydews that are intermittently ingested by the sandfly vector following a blood meal. ^13^C stable isotope labeling studies have shown that promastigotes exhibit a strong preference for sugars as carbon source and that the sugars are catabolized via glycolysis, the pentose phosphate pathways, succinate fermentation, and the tricarboxylic acid (TCA) cycle (18–20). These stages also coutilize nonessential amino acids during periods of high growth and can use amino acids as primary carbon source under low-glucose conditions (18–20). *Leishmania* amastigotes are metabolically less active than promastigotes, although they still exhibit a preference for sugars as carbon source, and are dependent on uptake and catabolism of sugars for survival in the host macrophages (21, 22). Strikingly, amastigotes exhibit reduced capacity to utilize nonessential amino acids but increased capacity to catabolize fatty acids (FAs) via β-oxidation to generate acetyl coenzyme A (acetyl-CoA) for the TCA cycle and anaplerotic pathways (18, 20, 22–24). This stage-specific switch in central metabolism is consistent with proteomic analyses of both axenic and lesion-derived amastigotes (25, 26). In particular, enzymes involved in degradation of unsaturated FAs by β-oxidation (abundant targets being monounsaturated and diunsaturated FAs) such as a putative 2,4-dienoyl-CoA reductase and trans-enoyl-CoA isomerase are significantly more abundant in amastigotes (26).

Here, we assessed the relevance of the single putative 2,4-dienoyl-CoA reductase (DECR) in L. major promastigote and amastigote stages. Phylogenetic analysis of DECR protein sequence revealed that this protein had been acquired by LGT early in the evolution of the trypanosomatids. Strikingly, this gene appears to have been retained in trypanosomatids that have evolved intracellular lifestyles (e.g., in genera *Leishmania*, *Trypanosoma*, and *Angomonas*) but has been lost in dixenous parasites with an exclusively extracellular lifestyle such as T. brucei. ^13^C-labeling studies of *decr*-deficient *Leishmania* parasites confirmed the activity of this enzyme. *In vitro* as well as *in vivo* infection experiments demonstrated that loss of *DECR* is associated with loss of virulence and the capacity to survive within host cells. Together, these data suggest that the acquisition and retention of this enzyme involved in β-oxidation of the abundant diunsaturated FAs have been critical in allowing *Leishmania* and other trypanosomatids to colonize intracellular niches.

## RESULTS

### *Leishmania* DECR is related to bacterial reductases.

**FIG 1:**
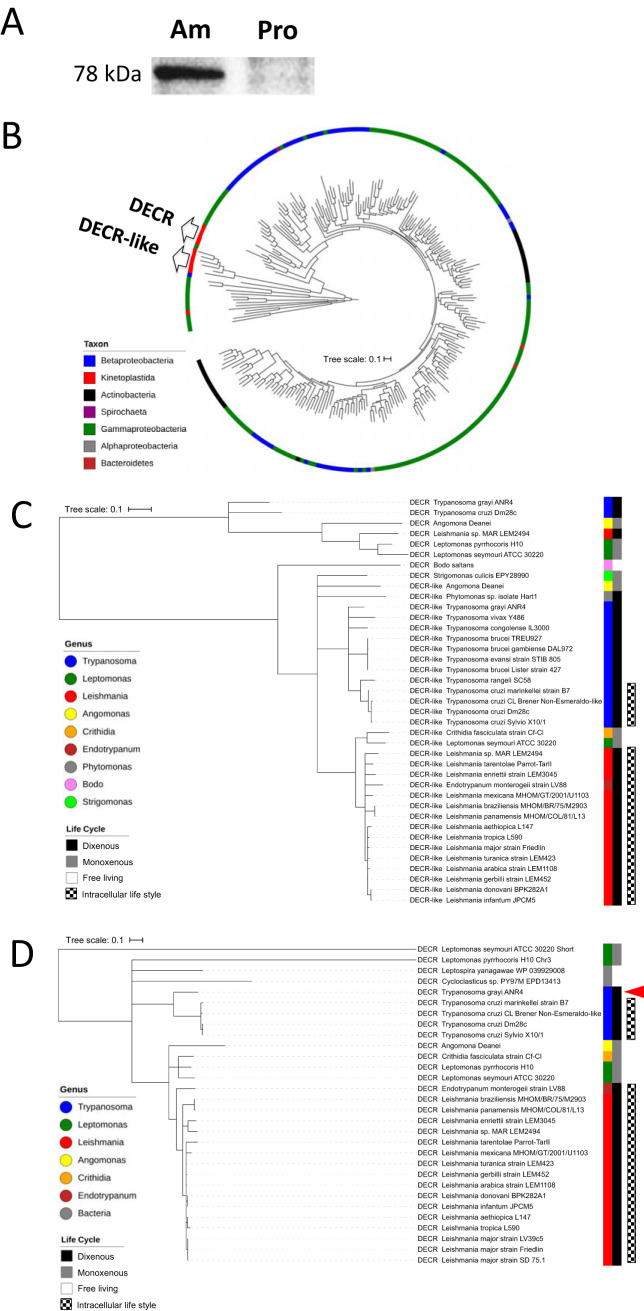
DECR expression in L. mexicana and phylogenetic analysis of bacterial and kinetoplastid DECR and DECR-like homologs. (A) Cell lysates (40 μg protein per lane) from axenic cultures of L. mexicana amastigotes (Am) and promastigotes (Pro) were immunoblotted with an anti-DECR serum. Equal levels of transfer were controlled by Ponceau S staining of the membrane before immune detection. (B) Overall circular phylogenetic tree for 248 DECR and DECR-like homologous protein sequences. Maximum likelihood analyses were performed using PhyML with the BEST tree search strategy. Branch robustness was assessed with Shimodaira-Hasegawa-like approximate likelihood ratio tests (SH-like aLRT), with a SH-like aLRT value of 0.995 supporting the branching off both the DECR and DECR-like sequences containing clades of their last common ancestor and with values of 1 (DECR-like protein) and 0.987 (DECR protein) supporting the branching off the last common ancestor shared with their respective nearest prokaryotic protein homologues. Sequences are colored according to taxonomic affiliation indicated in the legend. (C) Maximum likelihood phylogenetic tree of 33 kinetoplastid DECR-like protein sequences using 6 DECR sequences representative of relevant genera as outgroup. Monophyly of DECR-like protein sequences was indicated by SH-like aLRT branch support values of 0.96 or higher for the supraextant genus level. (D) Maximum likelihood phylogenetic tree of 26 kinetoplastid DECR routed by 4 outgroup sequences. Trees were constructed from multiple-sequence alignments followed by PhyML analysis. The horizontal tree scale bar represents 0.1 substitutions per amino acid (aa) site. The arrowhead in panel D indicates the dixenous extracellular trypanosomatid T. grayi.

Comparative proteomic analyses of Leishmania mexicana promastigotes and intracellular amastigotes previously revealed that several proteins associated with respiration, energy metabolism, and parasite stress responses are upregulated in amastigote stages (26). The data included several enzymes involved in FA metabolism, including the trifunctional enzyme alpha subunit (corresponding to *L. major* ortholog [LmjF26.1550]), putative 3-ketoacyl-CoA thiolase-like (LmjF31.1630), thiolase protein-like (LmjF31.1640), 3,2-trans-enoyl-CoA isomerase (LmjF31.2250), and DECR (LmjF33.0830) proteins (26). BLAST analysis indicated that most of the *Leishmania* enzymes involved in FA β-oxidation showed close sequence homology to equivalent enzymes in their vertebrate hosts. An exception was the DECR protein, which showed high homology to bacterial NADPH-dependent 2,4-dienoyl-CoA reductases and very limited sequence similarity to the equivalent human reductase (see Table S1 in the supplemental material). Consistent with previous proteomic analyses, we show here using DECR-specific antibodies that expression of DECR is indeed highly upregulated in amastigote stages (Fig. 1A).

10.1128/mBio.01057-20.5 TABLE S1 Sequence similarity search performed for L. major DECR using pBLAST. Download Table S1, XLSX file, 0.02 MB. Copyright © 2020 Semini et al. 2020 Semini et al. This content is distributed under the terms of the Creative Commons Attribution 4.0 International license.

To further define the origin of *Leishmania* DECR, we conducted a detailed phylogenetic analysis of related DECR protein sequences in other kinetoplastids, as well as in *Alphaproteobacteria*, *Betaproteobacteria*, *Gammaproteobacteria*, *Spirochaeta*, *Actinobacteria*, and *Bacteroidetes* species. Moreover, we included sequences encoding a protein that is of unknown function but is annotated as a DECR-like protein in kinetoplastids. Global phylogeny analysis of bacterial and kinetoplastid DECR homologs showed that all of the kinetoplastid sequences, except the shorter DECR sequence of Leptomonas seymouri, belonged to two clades, here referred to as the DECR and DECR-like clades (Fig. 1B). Remarkably, these two clades were not each other’s closest relatives. Instead, both formed robust groups with closer bacterial lineages (support values of 1 [DECR-like] and 0.987 [DECR] for branching off the ancestor shared with their respective nearest prokaryotic protein homologue and distinct summed-up branch lengths for DECR and DECR-like clades corresponding to their most recent common ancestors [MRCA], corresponding to 0.59 and 1.35 amino acid substitutions per site). This suggests that DECR and DECR-like proteins do not represent paralogs that arose by way of gene duplication but, more probably, represent the result of two events of independent LGT of genes of bacterial origin.

To further understand the phylogenetic relationships within the two major clades of kinetoplastid DECR homologs, we aligned all kinetoplastid DECR and DECR-like sequences with a selection of outgroup sequences (33 and 6 sequences [Fig. 1C] and 26 and 4 sequences [Fig. 1D], respectively). DECR-like sequences were present in all kinetoplastids (Fig. 1C), whereas DECR was detected in monoxenous kinetoplastids and in several dixenous kinetoplastids, which follow obligate intracellular lifestyles in their vertebrate hosts (Fig. 1D). Interestingly, DECR genes were absent from dixenous species, such as T. brucei, that colonize only extracellular niches in their respective hosts. The only exception to the latter finding was Trypanosoma grayi (indicated by an arrowhead in Fig. 1D), which has an exclusively extracellular lifestyle in its vertebrate (crocodile) host. However, the T. grayi DECR is predicted to be truncated and therefore to lack cofactor coordination residues at its N terminus, suggesting that it may not be functional (see Fig. S1 in the supplemental material).

10.1128/mBio.01057-20.1 FIG S1 Alignment of DECR protein sequences of L. major and T. grayi. Sequence alignment of L. major DECR (LmjF.33.0830) and T. grayi DECR (Tgr.72.1000) was performed using Geneious 10 software (cost matrix: BLSM62). Red box indicates N-terminal amino acids of L. major DECR that are absent in the T. grayi DECR protein sequence. Download FIG S1, EPS file, 1.5 MB. Copyright © 2020 Semini et al. 2020 Semini et al. This content is distributed under the terms of the Creative Commons Attribution 4.0 International license.

### The structure of *Leishmania* DECR is similar to those of bacterial reductases.

**FIG 2:**
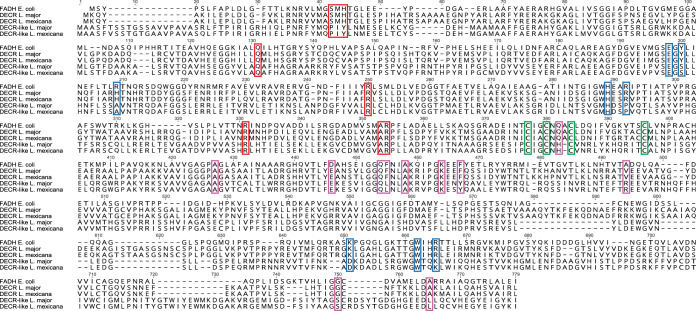
Sequence alignment of putative *Leishmania* DECR and DECR-like proteins. Sequence alignment of L. major DECR (LmjF.33.0830), L. mexicana DECR (LmxM.32.0830), L. major DECR-like (LmjF.06.0930), and L. mexicana DECR-like (LmxM.06.0930) proteins with E. coli FADH (AP_003630) was performed using Geneious 10 software (MUSCLE alignment). Amino acid residues involved in substrate binding as well as cofactor coordination were identified based on a crystal structure of E. coli FADH (27) and are highlighted with colored boxes. Red boxes, residues involved in FMN coordination; blue boxes, residues of the active site responsible in substrate binding; purple boxes, residues involved in FAD coordination; green boxes, residues involved in 4 Fe-4 S cluster coordination.

Since the leishmanial DECR orthologues are homologous to bacterial DECRs, we aligned the L. major and L. mexicana DECR proteins with Escherichia coli 2,4-dienoyl-CoA reductase (FADH). The structure of E. coli FADH, representing the only solved structure for a bacterial DECR (27), was previously shown to contain an iron-sulfur (4Fe-4S) cluster and noncovalently coordinated flavin mononucleotide (FMN) and flavin adenine dinucleotide (FAD) cofactors and was found to be functional as a monomer (27–31). The leishmanial DECR-E. coli FADH alignment revealed that all residues involved in the coordination of FAD, FMN, and the iron-sulfur cluster in the latter were conserved within the former sequences (Fig. 2). Residues involved in substrate binding as well as the catalytic residues (Tyr167 and His270) were also conserved. Most of the residues involved in NADPH binding and 10 of 11 residues involved in FAD coordination were located within the C-terminal 331 amino acids (Fig. 2).

Alignment of E. coli FADH, L. major, and L. mexicana DECR with their respective DECR-like proteins, which has been included in the phylogenetic analysis, showed that only 5 of 11 residues involved in FAD coordination, 7 of 9 residues involved in FMN coordination, and 5 of 9 residues in the active site were conserved in the latter (Fig. 2). This low match level was also found with DECR-like sequences of intracellular kinetoplastids (*Leishmania* species and Trypanosoma cruzi) and of extracellular kinetoplastids such as T. brucei and sequences from the free-living kinetoplastid Bodo saltans (Fig. S2).

10.1128/mBio.01057-20.2 FIG S2 Protein sequence alignment of DECR and DECR-like of kinetoplastids. Sequence alignment of L. major DECR (LmjF.33.0830), L. mexicana DECR (LmxM.32.0830), T. cruzi DECR (TCSYLVIO_007017), L. major DECR-like (LmjF.06.0930), L. mexicana DECR-like (LmxM.06.0930), T. cruzi DECR-like (TCSYLVIO_001867), T. brucei DECR-like (Tb927.7.5540), and B. saltans DECR-like (BSAL_12425) proteins with E. coli FADH (AP_003630) was performed using Geneious 10 software (MUSCLE alignment). Amino acid residues involved in substrate binding as well as cofactor coordination were identified based on a crystal structure of E. coli FADH (27) and are highlighted with colored boxes. Red boxes, residues involved in FMN coordination; blue boxes, residues of the active site responsible in substrate binding; purple boxes, residues involved in FAD coordination; green boxes, residues involved in 4 Fe-4 S cluster coordination. Download FIG S2, TIF file, 0.4 MB. Copyright © 2020 Semini et al. 2020 Semini et al. This content is distributed under the terms of the Creative Commons Attribution 4.0 International license.

**FIG 3:**
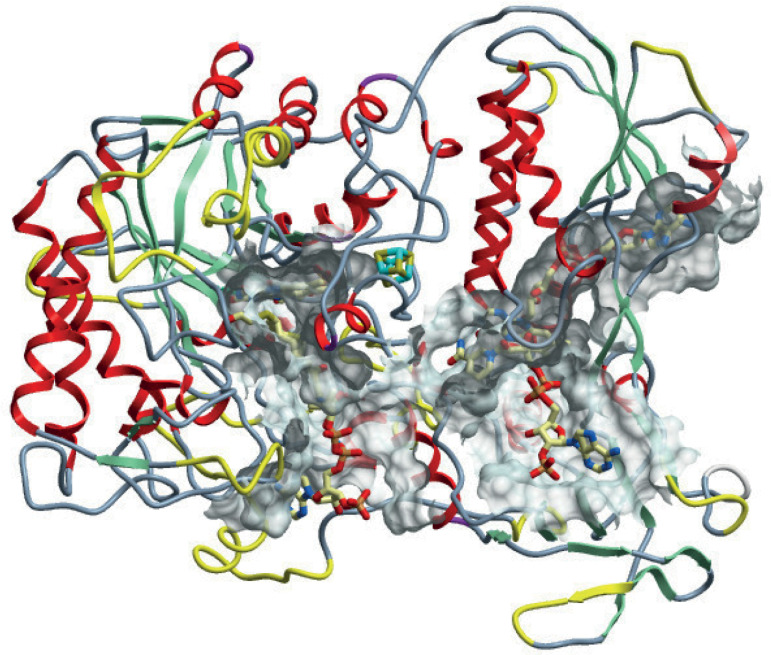
Model of L. mexicana DECR with differences from the E. coli FADH structure. Differences between L. mexicana DECR and E. coli FADH are highlighted in yellow. These are mainly in loop regions that resulted from insertions into the leishmanial DECR sequence. The solid mesh body represents contact area between protein and substrate.

To further predict the functionality of Leishmania DECRs, we modeled its three-dimensional (3D) structure against E. coli FADH using the online tool “Swiss-model” (https://swissmodel.expasy.org/). The models of L. major and L. mexicana DECR were subsequently recalculated and energy minimization was performed using UCSF Chimera (https://www.cgl.ucsf.edu/chimera/) and Amber force field (http://ambermd.org/AmberModels.php). *In silico* structure prediction revealed a remarkable overall similarity to E. coli FADH (Fig. 3). All relevant regions involved in FAD and FMN coordination, in NADPH binding, and formation of the iron-sulfur cluster as well as the active site were structurally conserved. Differences in the modeled leishmanial DECR structure resulted mainly from insertions in sequences localized in loops and helices (Fig. 3), which should not interfere with the predicted protein function. In contrast, modeling of L. major DECR-like protein suggested steric orientation differences that did not allow formation of an iron-sulfur cluster, even though all required cysteines were present.

### L. major DECR is the major reductase involved in polyunsaturated fatty acid β-oxidation.

**FIG 4:**
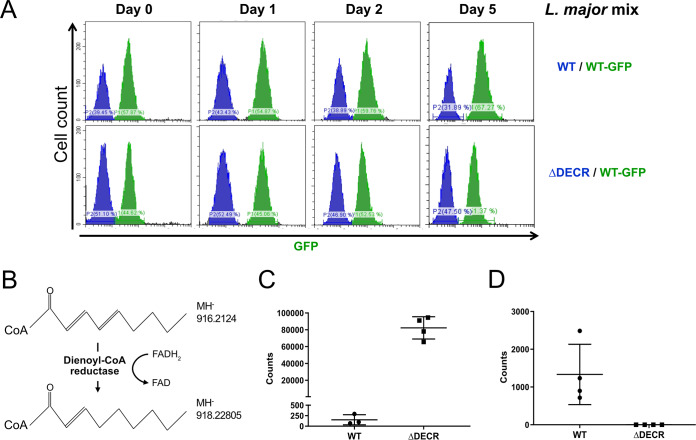
Loss of DECR does not affect promastigote growth *in vitro* but is associated with changes in intracellular levels of specific acyl-CoA species. (A) Competitive growth in the presence of a wild-type, GFP-expressing L. major was used to compare relative levels of fitness in one culture sampled at the indicated days over a 5-day observation time by flow cytometry. Cultures were initiated with mixes of 3 × 10^5^ parasites of each strain and reached densities of ∼10^7^ parasites per ml. (B) Linoleic acid-derived 2,4-decadienoyl-CoA is converted to 2-decaenoyl-CoA and by DECR (double bond position inferred based on the mode of action of E. coli FADH [27]). (C and D) LC/MS was used in selected ion monitoring mode to detect (C) 2,4-decadienoyl-CoA (*m*/*z* 916.21240) and (D) 2-decaenoyl-CoA (*m*/*z* 918.22805) in extracts from 2 × 10^8^ parasites per sample. Shown are data points corresponding to independent biological samples and their means with standard deviations (SD).

To investigate whether L. major DECR is a functional enzyme, L. major *DECR* null mutant parasites (L. major *Δdecr*) were generated by replacing the entire coding sequence of both genomic alleles with resistance markers through double homologous recombination. L. major Δ*decr* promastigotes were readily obtained and had a growth rate similar to that of wild-type (WT) promastigotes under standard culture conditions (Fig. 4A). To partly mimic amastigote growth conditions, promastigotes were also cultured at 33°C for 2 days in semidefined culture medium (SDM). Targeted liquid chromatography/mass spectrometry (LC/MS) analysis of total metabolite extracts of the thus-cultured wild-type and *Δdecr* L. major lines revealed a dramatic accumulation of 2,4-decadienoyl-CoA, one of the key precursors of DECR generated during the β-oxidation of linoleic acid, as well as the complete loss of the DECR product 3-decaenoyl-CoA (Fig. 4B to D).

**FIG 5:**
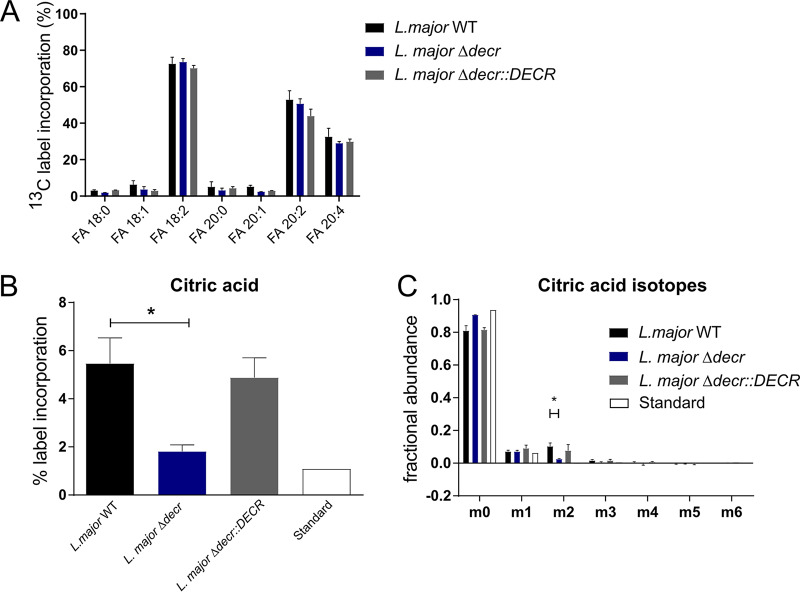
DECR is required for β-oxidation of linoleic acid *in vivo*. (A) L. major promastigotes were labeled with ^13^C-labeled linoleic acid and levels of selected ^13^C-FAs determined as methyl esters by GC/MS. ^13^C-labeled linoleic acid is incorporated into parasites. Shown are means with standard errors of the means (SEM) representing results from four, three, and two parasite cultures grown with WT, Δ*decr*, and Δ*decr*::*DECR* L. major, respectively. (B) L. major promastigotes were labeled with ^13^C-labeled linoleic acid and levels of ^13^C label incorporated into citric acid determined by GC/MS. Data represent means with SEM of results from four cultures (*, *P* < 0.05). (C) Analysis of citric acid mass isotopologues generated after ^13^C linoleic acid labeling. Data represent means with SEM of results from four, three, and two parasite cultures grown with WT, Δ*decr*, and Δ*decr*::*DECR* L. major, respectively (*, *P* < 0.05).

To further confirm the involvement of DECR in the β-oxidation pathway, parasites were incubated with ^13^C-labeled linoleic acid and the incorporation of ^13^C into intermediates in central carbon metabolism was monitored by gas chromatography/mass spectrometry (GC/MS). ^13^C-labeled linoleic acid was efficiently taken up by wild-type, *Δdecr*, and *Δdecr*::*DECR* promastigotes and partly elongated to other FA, as shown by the detection of ^13^C-labeled eicosaditraenoic and tetraenoic acids in all three parasite strains (Fig. 5A). In wild-type promastigotes, ^13^C-labeled linoleic acid was also catabolized by β-oxidation to acetyl-CoA as shown by the incorporation of ^13^C labeling in TCA cycle intermediates, such as citrate (Fig. 5B). The level of labeled citrate was repressed in the L. major *Δdecr* mutant and was restored in the complemented line (Fig. 5B). As shown previously (20), most of the C2 carbon backbones derived from FA β-oxidation appeared to exit the TCA cycle during the first cycle (possibly for generation of glutamate or malate). This was corroborated here by the specific generation of M+2 isotopomers of citrate and by the absence of isotopomers of larger mass (Fig. 5C). The M+2 isotopomers of citrate were reduced to levels that were close to background (natural abundance) levels in the L. major *Δdecr* mutant (Fig. 5C) consistent with DECR having a key role in β-oxidation of major unsaturated FAs.

### DECR is localized to mitochondria in *Leishmania*.

**FIG 6:**
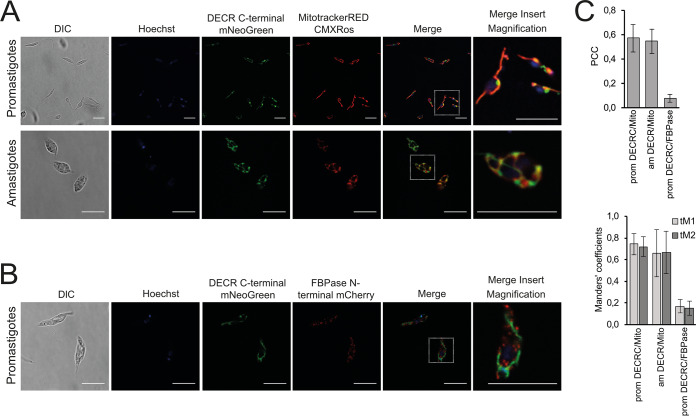
DECR localization in L. mexicana and L. major. (A) Live L. mexicana promastigotes and amastigotes expressing DECR-mNeoGreen were labeled with Hoechst (nuclei) and MitoTracker Red CMXRos (mitochondrion) and imaged by confocal microscopy. DIC, differential inference contrast. (B) L. mexicana promastigotes expressing DECR-mNeonGreen and FBPase-mCherry (glycosome marker) were fixed and imaged by confocal microscopy. Nuclei were stained with Hoechst. Scale bars: 10 μm. Scale bars for magnification: 2.5 μm. (C) Quantitation of colocalization. Pearson’s correlation coefficient (PCC) and Manders’ thresholded coefficients tM1 and tM2 were calculated using Costes’ automatic threshold regression on the deconvolved images. The data in the graphs present results from two independent experiments, each represented by several images (*n* ≥ 5). Error bars refer to standard deviations.

The *Leishmania* DECR-like protein has previously been localized to glycosomes that also contain other enzymes of FA metabolism (32), while the precise localization of DECR has not been determined (32–35). To localize the DECR protein, a C-terminal fusion protein containing mNeonGreen was expressed from the native gene loci. Direct fluorescence microscopy of live promastigotes expressing DECR-mNeonGreen showed that this protein is exclusively located in the mitochondria. In stationary-phase promastigotes, the mitochondrion has a distinct tubule structure that extends from the anterior flagellar pocket to near the posterior end of the cell (labeled with MitoTracker Red CMXRos in Fig. 6A). Interestingly, DECR-mNeonGreen was not uniformly distributed throughout the mitochondrion but was concentrated within a subdomain of the mitochondrion, which was invariably located proximal to the nuclear envelope (Hoechst staining). The identity of this subdomain remains undefined but might correspond to endoplasmic reticulum (ER)-mitochondrion junctions (or to mitochondrion-associated membranes [MAMs]) found in other eukaryotes. The DECR-mNeonGreen protein also colocalized with MitoTracker Red fluorescence in amastigotes (Fig. 6A). As with the promastigotes, there was some evidence of compartmentalization of DECR within the mitochondrion of amastigotes, although not to a single domain. No overlap was observed for the DECR-mNeonGreen protein and the glycosomal marker (mCherry–fructose-1,6-bisphosphatase [mCherry-FBPase]), indicating that DECR is not targeted to glycosomes (Fig. 6B). These visualizations and the results of their quantitative analysis (Fig. 6C) suggest that DECR is exclusively located to the mitochondrion in both major developmental parasite stages.

### DECR is a virulence factor in *Leishmania*.

**FIG 7:**
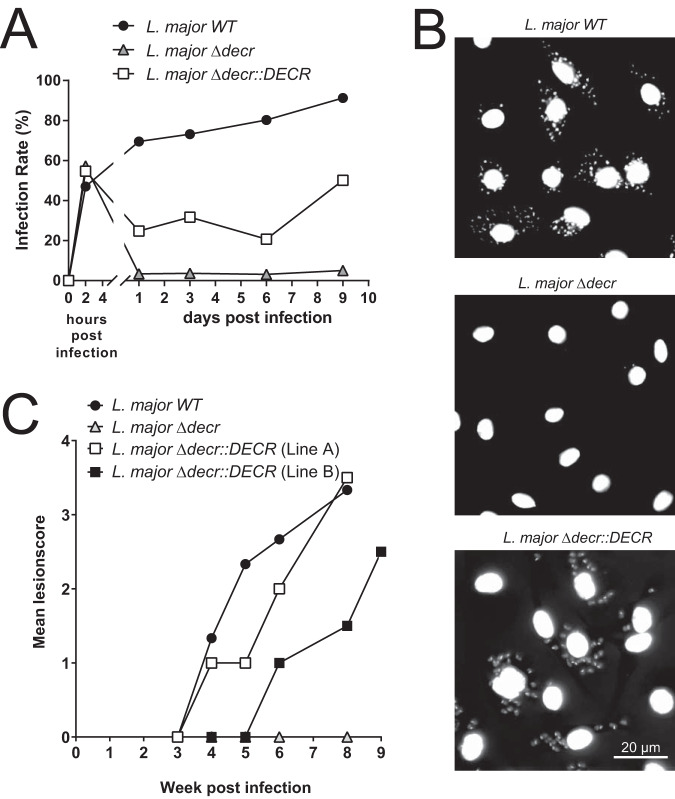
*In vitro* and *in vivo* infection assays. BMDMs were infected (multiplicity of infection [MOI] of 10) with stationary-phase wild-type (L. major WT), DECR-deficient (L. major Δ*decr*), or complemented DECR-deficient (L. major Δ*decr*::*DECR*) L. major promastigotes for 2 h. (A) Infection rate data are expressed as percentages of infected BMDMs and were determined by microscopy (at least 100 macrophages were counted). Data from one experiment representative of three is shown. (B) Infected BMDMs were washed to remove noninternalized parasites and further cultivated for 9 days. Cells were fixed with 4% paraformaldehyde (PFA), and DNA was stained using DAPI. (C) BALB/c mice were infected subcutaneously at the base of their tails with L. major WT (*n* = 3), L. major Δ*decr* (*n* = 3), and two independent L. major Δ*decr*::*DECR* complemented lines (both *n* = 2) (2 × 10^6^ promastigotes in the stationary phase of growth). Developing lesions were scored every week using the following lesion scores: 0, no swelling; 1, swelling or lesion size of <1 mm; 2, lesion size of 2 to 5 mm; 3, lesion size of 5 to 10 mm; 4, lesion size of >10 mm. Mice with lesion scores of more than 3 or 4 were euthanized. The average lesion score is indicated.

To determine whether DECR and β-oxidation of major diunsaturated FAs are required for intracellular growth and virulence, bone marrow-derived macrophages (BMDMs) were infected with L. major wild-type, mutant Δ*decr*, or complemented Δ*decr*::*DECR* parasites and growth was monitored for 9 days. All strains were taken up by BMDMs equally well (Fig. 7A). Mutant parasites lacking DECR were cleared over 24 h. In contrast, both wild-type and complemented L. major Δ*decr*::*DECR* parasites survived this initial period (Fig. 7A) and resumed growth over the day 9 postinfection observation period (Fig. 7A and B).

The virulence of the L. major *Δdecr* mutant was also assessed in the highly susceptible BALB/c mouse model. Mice were infected subcutaneously at the base of their tails, and lesion sizes were scored over a period of 8 weeks. Corroborating the infectivity results in BMDMs, L. major wild-type and L. major Δ*decr*::*DECR* parasites induced lesions that reached a size of 5 to 10 mm (lesion score 3), at which point animals had to be euthanized. In contrast, L. major Δ*decr* parasites failed to develop quantifiable lesions (lesion score 0; Fig. 7C). However, as often observed with attenuated *Leishmania* mutant lines (36–38), limiting dilution culturing from tissues at the site of injection revealed that some L. major Δ*decr* parasites persisted 8 weeks post *in vivo* infection. Thus, DECR is a virulence factor and *decr*-deficient parasites were unable to cause lesions in a highly susceptible host.

## DISCUSSION

In the present study, we characterized the function of DECR, an enzyme that is highly upregulated in intracellular pathogenic stages of *Leishmania* and involved in the β-oxidation of unsaturated FAs. Phylogenetic analyses suggested that DECR had been acquired by LGT from a bacterial donor early during the evolution of kinetoplastids, adding to the pathway of FA β-oxidation in mitochondria. The results of our genetic and biochemical studies suggest that DECR is the major 2,4-dienoyl-CoA reductase in these parasites and essential for intracellular parasite growth and virulence in animal models, highlighting the importance of FA catabolism for virulence.

In the last 4 decades, the structure and activity of both peroxisomal and mitochondrial eukaryotic DECR enzymes have been extensively characterized (39–43) (for reviews, see references 44 and 45). Moreover, it has been observed that DECR deficiency is associated with severe disorders in mice and humans (46–48). To date, the investigation of prokaryotic and eukaryotic DECR function has been restricted to model organisms such as E. coli and Saccharomyces cerevisiae (27, 29–31, 49). Although the relevance of lipid metabolism in trypanosomatids has been broadly studied (50–52), the β-oxidation pathway of major unsaturated FAs in these organisms has received little attention. In trypanosomatid genomes, two genes are annotated as encoding proteins with homology to bacterial DECR, the putative DECR and DECR-like proteins.

Our *in silico* analyses demonstrated that the *Leishmania* and T. cruzi DECR proteins retain the same structure and residues involved in cofactor coordination and substrate binding as the E. coli DECR ortholog FADH. These analyses indicated that trypanosomatid DECRs are active reductases. This was experimentally confirmed by targeted metabolomic analysis of the *Leishmania* Δ*decr* mutant, which showed that a major substrate of DECR, 2,4-decadienoyl-CoA, accumulated in the absence of the enzyme. In contrast, structural predictions for *decr-like* gene-encoded proteins indicated that they may not bind required cofactors due to significant differences in amino acid residues involved in their coordination and are likely not contributing to reduction of 2,4-decadienoyl-CoA by the parasites. Divergences between DECR and DECR-like proteins also extend to their localization. The DECR-like protein is thought to be localized to the glycosomes of *Leishmania* and *Trypanosoma* species (33–35). In contrast, we show here that the *Leishmania* DECR protein is localized to the mitochondrion. Interestingly, results of direct fluorescence microscopy imaging of live promastigotes suggested that the DECR-mNeonGreen reporter protein was concentrated in a subdomain of the mitochondrion juxtaposed to the ER-nuclear envelope. Similar junctions between the mitochondria and ER occur in other eukaryotes and are associated with intraorganellar transport of phospholipid precursors (53). Whether DECR is concentrated within these MAM domains and the functional significance of this additional compartmentalization need to be investigated.

DECR has been transferred to kinetoplastids, likely from proteobacteria by LGT (10, 15). Extant *Leishmania* genomes have been significantly affected by prokaryote-to-eukaryote LGT, with 0.96% of the protein-coding genes originating from LGT events, the highest percentage among microbial parasites (10). The relevance of LGT for the development of an intracellular life cycle has recently been highlighted by the description of the acquisition and subsequent evolution of a family of dual-activity glycosyltransferase-phosphorylases in *Leishmania* spp. (54). With respect to DECR, previous studies on LGT in trypanosomatids did not differentiate between DECR and DECR-like proteins. However, in doing so, we propose that these genes were acquired in two independent events and that extracellular parasites such as the African trypanosomes may have lost DECR subsequently. This gene loss event accounts for an evolutionary path of the DECR locus that is consistent with the evolution of this family of organisms.

Acquisition by separate LGT events rather than by gene duplication and diversification is most consistent with the results of our analysis. Accordingly, a DECR-like gene was acquired first in an ancestral kinetoplastid and DECR gene transfer happened most likely after the divergence of free-living and parasitic kinetoplastids. However, the description of this scenario needs a cautionary note, since (i) the estimates of the 95% highest posterior densities of the most recent common ancestors (MRCA) overlapped (i.e., the order of the transfer events was not firmly ascertained, although our Bayesian Monte Carlo Markov chain [BMCMC] analyses indicated a more recent MRCA for kinetoplastid DECR protein than for kinetoplastid DECR-like protein), (ii) detection of the DECR gene in B. saltans genome might have failed or it might have been lost secondarily in this lineage, and (iii) the bacterial group(s) at the origin of the DECR and DECR-like LGTs could not be resolved based on current knowledge. Nevertheless, the kinetoplastid DECR and DECR-like gene orthologs separated from clearly different closest related bacterial lineages.

*DECR* loss in nonintracellular trypanosomes appears to have occurred independently in several extracellular trypanosomes, and this could be related to the stability of the respective genomic regions of the DECR and DECR-like genes (see TriTrypDB [http://tritrypdb.org/tritrypdb/]). The cistronic regions upstream of *DECR* loci are characterized by the presence of β-tubulin genes. Synteny in this region is less conserved among the sequenced genomes (see, e.g., https://tritrypdb.org/tritrypdb/app/record/gene/LmjF.33.0830#SyntenyGbrowseUrl) than for the respective region upstream of *DECR-like* genes, indicating that the DECR locus is in a region prone to frequent genomic rearrangements. Rearrangement of this region would also explain the situation found in T. grayi, which possesses only a truncated version of the *DECR* gene encoding a likely nonfunctional, N-terminally shortened protein. Loss of DECR in extracellular trypanosomes corroborates the existence of selective pressure acting on intracellular trypanosomatids to maintain DECR gene integrity and hence enzyme activity for survival and growth.

Indeed, genome data revealed that DECR is present in trypanosomatids that have the capacity to survive intracellularly. Of particular note is the presence of *DECR* genes in monoxenous kinetoplastids (Angomonas deanei, *Crithidia* spp., *Leptomonas* spp.). For example, A. deanei, previously named Crithidia deanei, was shown to be infective to, in particular, dexamethasone-immunosuppressed BALB/c mice (55, 56). Parasites related to Crithidia fasciculata (57) and L. seymouri, respectively, were identified as coinfecting or sole agents in immunocompromised HIV patients diagnosed with leishmaniasis (58–60). The clinical cases show that monoxenous trypanosomatids of genera or species that are normally considered nonpathogenic can survive within vertebrate hosts and host cells, in particular, in immunocompromised hosts (57, 59, 61–63).

Genetic deletion of DECR in *Leishmania* led to a loss of virulence in both macrophage and animal infection models. There are a number of possible explanations for loss of virulence. First, it is possible that intracellular amastigotes are dependent on FA as a major carbon source. In particular, β-oxidation of major unsaturated FA species may be required for production of acetyl-CoA in the mitochondrion and for anaplerotic production of citrate and glutamate via the initial steps in the oxidative TCA cycle (19, 20). *De novo* synthesis of glutamate appears to be essential in amastigotes, which have a greatly reduced capacity to take up nonessential amino acids (20). However, while our metabolomic analyses of parasites labeled for extended times showed that loss of DECR leads to a complete block in final steps in unsaturated FA β-oxidation, the mutant may still be able to generate minor amounts of acetyl-CoA during the non-DECR-dependent cycles of this process. Interestingly, the accumulation of DECR substrates in mammalian cells has been shown to inhibit β-oxidation of other FA (64), resulting in a global defect in FA β-oxidation. While our results are consistent with a complete block of β-oxidation in the DECR mutant, this will have to be investigated in future studies.

Alternatively, or in addition, DECR may be required to prevent accumulation of toxic levels of unsaturated FAs in intracellular parasite stages. Free FAs are evolutionarily ancient innate immunity effector molecules with potent microbicidal activity and play a prominent role in defense in organisms from mollusks to humans (65, 66). In general, unsaturated FAs exhibit greater antimicrobial activity than saturated FAs and unsaturated FAs with double bonds in *cis* orientation are more toxic than those with double bonds in *trans* orientation (67, 68). Cell lysis, lipid peroxidation, and impairment of nutrient uptake are thought to be toxic consequences of the effects of FAs on bacteria (69, 70).

Free FAs are also cytotoxic to eukaryotic cells such as S. cerevisiae (71–73) and to pathogenic protozoa such as *Plasmodium* spp. *in vivo* and *in vitro* (74, 75). Significantly, the intracellular parasite Toxoplasma gondii presumably lacks a functional β-oxidation pathway but has retained a DECR protein of the eukaryotic peroxisomal family (see Toxoplasma gondii ME49_226300[TGME49_226300] in ToxoDB [https://toxodb.org/toxo/app/record/gene/TGME49_226300]). T. gondii tachyzoites readily take up host FAs (76), but supplementary addition of unsaturated FAs (not saturated FAs) was previously shown to impair parasite growth and replication in host fibroblasts (77). However, we have found that neither the wild-type nor DECR-deficient L. major parasites are sensitive to physiologically relevant concentrations of free FA. Specifically, a 5-fold excess over the usual cell culture levels of free linoleic acid did not affect *in vitro* growth (see Fig. S3 in the supplemental material).

10.1128/mBio.01057-20.3 FIG S3 DECR deficiency has no consequences for *in vitro* growth of L. major parasites in the presence of a high abundance of unsaturated FA. Parasite cultures were first adapted and further cultivated in completely defined medium (CDM) containing 0.75% essentially FA-free BSA supplemented with 100 U/ml penicillin and 0.1 mg/ml streptomycin. To assay the effect of exogenously added FFA on DECR-dependent growth, oleic acid (OA) or linoleic acid (LA) was added at 150 μM to CDM lacking glucose and growth of L. major wild-type, DECR-deficient mutant, and DECR-complemented mutant parasites at 33°C and pH 5.5 was monitored over 3 days. Download FIG S3, TIF file, 0.3 MB. Copyright © 2020 Semini et al. 2020 Semini et al. This content is distributed under the terms of the Creative Commons Attribution 4.0 International license.

Lack of DECR could also have led to reductive stress in mitochondria, thereby affecting viability. However, the mutants were not different from the parental wild-type parasites when exposed to increasing concentrations of dithiothreitol (DTT) or N-acetyl cysteine, commonly used to assess susceptibility to reductive stress (see Fig. S4).

10.1128/mBio.01057-20.4 FIG S4 Unaltered sensitivity of L. major wild-type (wt), *decr* mutant, and complemented strains to reductive stress induced by DTT or N-acetyl cysteine (NAC). Parasites were cultured in SDM supplemented with the indicated range of concentrations of DTT (left) or NAC (right) covering the range of nontoxic to toxic amounts. Growth was monitored over 3 days by turbidimetry. (*n* = 4 independent repeats; error bars refer to standard deviations [SD]). Download FIG S4, TIF file, 0.5 MB. Copyright © 2020 Semini et al. 2020 Semini et al. This content is distributed under the terms of the Creative Commons Attribution 4.0 International license.

Finally, it is possible that intracellular amastigotes may be highly sensitive to accumulation of DECR substrates, such as 2,4-decadienoyl-CoA. While the accumulation of this intermediate was not lethal to promastigote stages, it was able to lead to dysregulation of mitochondrial function and production of additional oxidative stress that is lethal in the intramacrophage niche (20). As said above, accumulation of DECR substrates in mammalian cells (64) led to a global halt of β-oxidation.

In summary, we show that the acquisition of a mitochondrially located homologue of a DECR gene by early trypanosomatids via LGT is likely to have enhanced the metabolic fitness of these protists in their insect vectors and subsequently to have allowed several medically important pathogens to evolve intracellular lifestyles in their mammalian hosts. The bacterial origin of the DECR homologues could be exploited in the development of specific antileishmanial therapies.

## MATERIALS AND METHODS

### Growth of *Leishmania* parasites.

L. mexicana *mexicana* (MNYC/BZ/62/M379) expressing DsRed (78) and wild-type L. major (WHOM/IR/-/173) (L. major WT) (79) as well as *decr*-deficient L. major (L. major Δ*decr*) and *decr*-complemented L. major (L. major *Δdecr*::*DECR*) strains were cultured in semidefined culture medium (SDM) supplemented with 10% heat-inactivated fetal bovine serum (FBS), 0.1 mM adenine, 1 μg/ml biotin, 5 μg/ml hemin, and 2 μg/ml biopterin (all from Sigma-Aldrich, Steinheim, Germany). For metabolic analyses, L. major parasites were adapted and further cultivated in completely defined medium (CDM [80]) containing 0.75% essentially FA-free bovine serum albumin (BSA) (Sigma-Aldrich, Steinheim, Germany) supplemented with 100 U/ml penicillin and 0.1 mg/ml streptomycin (Capricorn Scientific, Ebsdorfergrund, Germany). Promastigotes were incubated at 25.5°C with 5% CO_2_. Differentiation of L. mexicana promastigotes to amastigotes in axenic culture was carried out as described previously (26). Briefly, stationary promastigotes were diluted 1 to 10 in Schneider’s *Drosophila* medium (Sigma-Aldrich, Steinheim, Germany) supplemented with 20% heat-inactivated fetal bovine serum (Gibco, Paisley, United Kingdom) and adjusted to pH 5.5 and were incubated for 1 week at 33°C until differentiation into amastigotes was accomplished.

### Generation of *decr*-deficient and complemented Leishmania major.

In order to generate *DECR* deletion mutants, the 5′-flanking and 3′-flanking regions of *DECR* of L. major were amplified from genomic DNA (gDNA) and ligated into the pUC18 vector together with either the hygromycin resistance gene or the neomycin resistance gene. A HindIII-linked 5′ primer (5′-GCTAAGCTTGAGTCTTGTCAGTCCTTCTTG-3′) and a SpeI/XbaI-linked 3′ primer (5′-GCTTCTAGACTAGTTTTTGTGCAATTTCGTTGCCG-3′) were used for the 5′-flanking region and a BamHI-linked 5′ primer (5′-GCTGGATCCGGGTCGACTAGGTTGCCGTCAC-3′) and a HpaI/KpnI-linked 3′ primer (5′-GCTGGTACCGTTAACGGCACCAGCACACCACAGCA-3′) were used for the 3′-flanking region for the amplification of flanking regions necessary for the homologous recombination. The 5′-flanking region was subjected to HindIII and XbaI digestion and introduced into the HindIII/XbaI cut of the vector. The hygromycin resistance gene was subjected to SpeI and BamHI digestion and introduced into the SpeI/BamHI cut. The 3′-flanking region was subjected to KpnI and BamHI digestion and introduced into the KpnI/BamHI cut of the plasmid (for better understanding of the following cloning strategy, this construct is referred to here as plasmid A). In order to obtain the final plasmid containing neomycin as a selection marker, the hygromycin resistance gene was replaced by the neomycin resistance gene using both digestion with plasmid A and insertion with SpeI and BamHI. Every cloning step was verified by restriction digestion and sequencing. In order to obtain fragments for homologous recombination, flanking regions together with the resistance gene were excised from the generated plasmids using MluI and HpaI.

The strategy for the generation of a complemented strain consisted in the reinsertion of the *DECR* gene at its original locus in L. major *decr*-deficient parasites. Therefore, hygromycin or neomycin resistance genes were replaced by bleomycin resistance genes for selection. This resistance gene was amplified from a previously constructed unpublished vector containing bleomycin resistance gene between the two above-mentioned *DECR*-flanking regions. For this purpose, a SpeI-linked 5′ primer (5′-CGGACTAGTCATGGCCAAGTTGACC-3′) and an XbaI-linked 3′ primer (5′-CGGTCTAGAGGTCAGTCCTGCTCCT-3′) were used. The fragment was then subjected to SpeI and XbaI digestion and introduced into the SpeI/XbaI cut of pSSU-int vector, which contains the intergenic region of the cysteine proteinase B (CPB) 2.8 gene cluster of L. mexicana (CPB 2.8 inverted repeat [IR] [81]), replacing the original hygromycin resistance gene. The resulting plasmid (called plasmid B) was then subjected to SpeI and KpnI digestion, and the isolated bleomycin-CPB 2.8 IR fragment was inserted in plasmid A, which had previously been subjected to SpeI and KpnI digestion, in order to remove the hygromycin resistance gene and 3′ flanking region. The resulting plasmid (called plasmid C) contained the 5´flanking region, bleomycin resistance gene, and CPB 2.8 IR region. The *decr* gene and its 3′ flanking region were amplified from L. major gDNA using a KpnI-linked 5′ primer (5′-CGGGGTACCATGAAGCAGTACGCAAAGAT-3′) and a KpnI-linked 3′ primer (5′-CGGGCTCTTTCGCTTGCCGATCACAACCGTC-3′). The fragment was digested with KpnI and introduced by nondirectional cloning into KpnI cut downstream of the CPB 2.8 IR region of plasmid C. Orientation of the different fragments in the final construct was checked by asymmetric restriction digestion and sequencing, and the results were linearized using AseI/HindIII for transfection experiments.

### Transfection of L. major.

Promastigotes (2 × 10^7^) in the logarithmic-growth phase were transfected with 3 to 5 μg DNA by electroporation using a human T cell Nucleofector kit (Lonza, Basel, Switzerland). After 24 h of culture in SDM, selection pressure was applied by supplementing the medium with 16 μg/ml hygromycin B (Carl Roth, Karlsruhe, Germany), 16 μg/ml G418 (Carl Roth, Karlsruhe, Germany), or 40 μg/ml bleomycin (InvivoGen, San Diego, CA, USA). After 1 week, cultures in 48-well plates were analyzed for proliferating parasites. These were passaged three times until a line was considered stable. Genomic DNA was obtained and homologous recombination was verified by PCR and sequencing. Knockout of DECR of both copies was checked and the possibility of retention of an extra copy of chromosome 33 in double knockout parasites excluded using Southern blot analyses and DECR ORF-specific PCR and Western blot analysis using a DECR-specific antiserum (see below).

### DECR data sets and multiple-sequence alignments.

We gathered 10,561 bacterial and kinetoplastid DECR/NADPH-dependent DECR protein sequences from public databases (TriTrypDB for DECR and DECR-like protein sequences of trypanosomes; NCBI integrated in Geneious for DECR and NADPH-dependent DECR protein sequences of betaproteobacteria and gammaproteobacteria, as well as for DECR protein sequences of *Marinobacter* and *Corynebacterium*) and from BLAST-P analysis performed using the DECR protein sequences of Bodo saltans and L. major as query sequences, which we aligned using MAFFT (82). We selected conserved alignment blocks using gblocks (83) as implemented in SeaView v4 (84) and then used FaBox v1.41 (85) to identify the 3,092 unique sequences in the alignments of the resulting 510 amino acid positions.

To investigate the deep evolutionary history and the origins of kinetoplastid homologs of DECR, we reduced this data set to sequences exhibiting at least 20% divergence with T-Coffee (86). The final alignment comprised 248 sequences, including 12 sequences derived from kinetoplastids.

### Phylogenetic analyses.

We used Prottest v3.2 to perform model selection on the three alignments, using a full maximum likelihood (ML) optimization and the Bayesian information criterion for model comparison (87); in all cases, the selected model was LG+I+G+F. We then ran phylogenetic analyses in ML (for all alignments) and Bayesian frameworks (for the first alignment).

ML analyses were performed using PhyML v3 (88) with the full optimization and BEST tree search strategy and assessing branch robustness with Shimodaira-Hasegawa-like approximate likelihood ratio tests (SH-like aLRT) (89). The resulting tree was rooted using Tempest by minimizing the variance of root-to-tip distances (90).

For the first alignment, we also ran multiple Bayesian Monte Carlo Markov chain (BMCMC) runs with BEAST v1.8.2 (91), using a lognormal relaxed clock and a birth-death speciation model for tree shape. We checked run convergence and appropriate sampling of the posterior (with effective sample sizes being >200) using Tracer v1.6 (http://tree.bio.ed.ac.uk/software/tracer/), combined different run outputs using LogCombiner v1.8.2. (distributed with BEAST), and summarized the posterior set of trees (PST) on the maximum clade credibility tree using TreeAnnotator v1.8.2 (distributed with BEAST). Branch robustness was assessed with posterior probabilities. Node depths (expressed as numbers of amino acid substitutions per site) of the most recent common ancestors (MRCA) of kinetoplastid *DECR* and *DECR*-like sequences were extracted from the PST.

### LC/MS analysis of parasite metabolites.

L. major strains were cultivated in completely defined medium (CDM) containing 5 mM glutamate until they reached the late logarithmic phase. Parasites were harvested by centrifugation, washed once with phosphate-buffered saline (PBS), and resuspended in glucose-free CDM supplemented with ^13^C-labeled linoleic acid (Cambridge Isotopes Laboratories, Andover, MA, USA) and 5 mM glutamate at a density of 0.75 × 10^6^ parasites/ml. Parasites were incubated at 33°C for 24 h and quenched in the late logarithmic phase in a dry-ice-cooled ethanol bath and harvested by centrifugation and washed thrice in cold PBS before further processing and extraction of metabolites.

We used a modified version of a previously reported untargeted LC/MS workflow (92) for our targeted LC/MS analysis of the main DECR product and substrate. For extraction, the cell pellets were dissolved in acetonitrile/water (8:2 [vol/vol]) by sonication in a water bath and insoluble material was separated by centrifugation (21,500 × *g*, 10 min, 0°C). Samples (10 μl) were analyzed on a Thermo Fisher Vanquish ultra-high-pressure liquid chromatograph coupled to a Thermo Q Exactive mass spectrometer. Metabolites were separated on a SeQuant pHILIC column (450 by 4.5 mm with 5-μm pore size). The chromatographic gradient used started with solvent A (80% AcN) and solvent B (20% 20 mM NH_4_HCO_3_) for 1 min. Solvent B was linearly increased to 80% over 18 min and further to 95% over 2 min. The column was equilibrated to starting conditions for 4.5 min before the next sample was injected. Samples were run in a randomized order with regular blanks and pooled biological controls. Metabolites were ionized in a heated electrospray H-EASY II ion source in negative mode at 3.5 kV. Nitrogen gas flow parameters were set to 40 for sheath gas, 5 for auxiliary (aux) gas, and 0 for sweep gas. The transfer capillary was heated to 275°C and the S-lens radio frequency (RF) level set to 55. The mass spectrometer was calibrated in negative-ionization and positive-ionization modes immediately before analysis was performed using the corresponding Calmix reagents (Thermo Fisher, Waltham, MA, USA) and pyruvic acid. The mass spectrometer was operated in selective ion monitoring mode with 0.5-atomic-mass-unit (amu) mass isolation windows. Data were analyzed manually using the Qual Brower in Thermo Xcalibur 4.0.27.19 software. 2,4-Cecadienoyl-CoA and 2-decaenoyl-CoA were identified based on the exact mass within a 5-ppm mass window, the expected isotope distribution, and a mass shift by 10.03 of 2,4-decadienoyl-CoA in ^13^C-labeled linoleic acid-fed parasites. Metabolites were quantified in counts from extracts of 2 × 10^8^ cells per sample.

### Analysis of polar and apolar metabolites by GC/MS.

GC/MS analysis of extracts was performed as previously described (20) with a modified quenching procedure. Parasites were quenched by dilution into excess ice-cold PBS, washed two times in PBS, frozen in liquid nitrogen, and stored at –70°C for further analysis. The pellet was extracted in chloroform/methanol/water (1:1:3 [vol/vol]), and the supernatant extracts were made biphasic by the addition of water containing 1 nmol internal standard *scyllo*-inositol (final chloroform/methanol/water ratio, 1:2:1.4 [vol/vol]). Polar metabolites were dried and methoxylated using 20 mg/ml methoxyamine–pyridine at room temperature for 16 h and trimethylsilylated in BSTFA/TMCS (Sigma-Aldrich, Steinheim, Germany) for 1 h at room temperature before being analyzed on an Agilent 7890A-5975C GC/MS system (Agilent, Santa Clara, CA, USA) equipped with a 30-m DB5 column. Fatty acids were analyzed as their corresponding methyl esters that were generated after methanolysis of the dried chloroform phase in 0.5 M methanolic acid–HCl at 80°C overnight under a partial vacuum. The data were analyzed using ChemStation software (version D.01.02.16; Agilent) and DExSI (93).

### Infection of mice and bone marrow-derived macrophages.

All animal experiments were approved by an ethics committee and licensed by the legal authorities under the license PPL 60/03581 (United Kingdom), and the licenses T 0249/14 and H 0101/12 (Germany) to T. Aebischer. BALB/c and C57BL/6 mice were purchased from Charles River, Sulzfeld, Germany, and maintained in a conventional animal facility. For *in vivo* infection experiments, mice were infected with 4 × 10^6^ stationary-phase promastigotes at the base of the tail where lesions developed. Developing lesions were scored every week (94), mice were killed by cervical dislocation at the end of the experiment, and lesion tissue was excised for parasite isolation and further cultivation in axenic cultures. For *in vitro* infection studies, macrophages were differentiated from bone marrow of 6-to-8-week-old female mice as described previously (95) and were infected with axenic promastigotes at a multiplicity of infection of 10 for 2 h at 33°C with 5% CO_2_. Before infection, promastigotes in the stationary phase were diluted 1:3 in Schneider’s *Drosophila* medium (Sigma-Aldrich, Steinheim, Germany) supplemented with 20% heat-inactivated fetal bovine serum (Gibco Paisley, United Kingdom) and with MES [2-(n-morpholino)ethanesulfonic acid] adjusted to pH 5.5 for 24 h at 33°C. Infected cells were incubated for the indicated time points at 33°C and 5% CO_2_.

### SDS-PAGE and Western blotting.

Proteins were separated by the use of 10% SDS-PAGE (Bio-Rad, Munich, Germany) and transferred to a nitrocellulose membrane using a semidry blotting system (Hoefer Inc., Holliston, MA, USA). The protein transfer on the membrane was verified by staining with Ponceau S solution, followed by decoloration with 0.1% acetic acid solution. Membranes were blocked with 5% BSA dissolved in PBS (pH 7.3) containing 0.1% Tween 20 (PBS-T). In order to reduce unspecific binding, anti-DECR serum (1:20,000 dilution) was preincubated for 30 min in blocking buffer containing 25% cell lysate of *decr*-deficient parasites. Anti-DECR serum was generated by immunizing rabbits (Preclinics, Potsdam, Germany) with three peptides corresponding to epitopes predicted to be located on the surface of leishmanial DECR that were distinct from the DECR-like gene products. Secondary anti-rabbit antibody was diluted in blocking buffer, and, after the membrane was washed twice with PBS-T and once with PBS, a chemiluminescent reaction was carried out using SuperSignal West Dura (Thermo Fisher Scientific, Darmstadt, Germany) and data were digitally acquired with a Bioimager.

### Subcellular localization of DECR.

DECR was C-terminally tagged with mNeonGreen using CRISPR/Cas9 (96) (http://www.leishgedit.net). A L. mexicana (MNYC/BZ/62/M379) line constitutively expressing Cas9 and T7 RNA polymerase (RNAP) was created after transfection with pTB007 plasmid (L. mexicana T7Cas9) and was routinely maintained in RPMI 1640–10% FBS containing 50 mg/ml hygromycin B Gold (Invitrogen, Australia). The T7Cas9 line was transfected with small guide DNA (sgDNA) and donor plasmid DNA, specific for the gene of interest. DECR-3′ sgDNA was generated by PCR using 3′sgFP (GAAATTAATACGACTCACTATAGGAGCACTGCGTCGTACTGTGAGTTTTAGAGCTAGAAATAGC) and the universal sg-RP (AAAAGCACCGACTCGGTGCCACTTTTTCAAGTTGATAACGGACTAGCCTTATTTTAACTTGCTATTTCTAGCTCTAAAAC) and High Fidelity Phusion polymerase (New England Biolabs, Australia) in a 25-μl volume containing 0.2 mM deoxynucleoside triphosphates (dNTPs) and 2 μM primers.

DECR donor DNA was amplified from plasmid pPLOTv1 Neo-mNeonGreen (96) with DECR-DFP (CTGCAAGCTCATAGCGTGGCGATACGTCTGGGTTCTGGTAGTGGTTCCGG) and DECR-DRP (AAGAGGAAGAGACATCACGCTTGGCCCGCCCCAATTTGAGAGACCTGTGC) using High Fidelity Phusion polymerase (0.2 mM dNTPs, 0.5 μM primers, 2 U polymerase, 30 ng plasmid DNA, 1 M betaine [Sigma-Aldrich, Australia]) All primers were designed using the software tool at http://www.leishgedit.net.

The glycosomal marker fructose-1,6-bisphosphatase (FBPase) was N-terminally tagged with mCherry (pPLOTv1 puro-mCherry) (97). FBPase-5′sgDNA was produced with 5′sgFP (GAAATTAATACGACTCACTATAGGGGCTGCGGTAGGAGACCTGAGTTTTAGAGCTAGAAATAGC) and universal s-RP (AAAAGCACCGACTCGGTGCCACTTTTTCAAGTTGATAACGGACTAGCCTTATTTTAACTTGCTATTTCTAGCTCTAAAAC), using the same PCR conditions as described above. FBPase donor DNA was amplified from plasmid pPLOTv1 puro-mCherry (96) (http://www.leishgedit.net) with FBPase-UFP (CAACCCCCCCCCCCTTTCCACGTTCAACCCGTATAATGCAGACCTGCTGC) and FBPase-URP (AGTGGGAGTGGGGGTGCGCCTGATGTCCATACTACCCGATCCTGATCCAG) using the same conditions as described above.

L. mexicana T7Cas9 promastigotes (mid-log phase, 4 × 10^7^ cells/transfection) were suspended in chilled electroporation buffer (EPB; 21 mM HEPES, 137 mM NaCl, 5 mM KCl, 6 mM glucose, 0.7 mM Na_2_PO_4_, pH 7.4), in a 4-mm-path-length cuvette and electroporated with purified sgDNA and donor DNA (combined in a 30-μl volume) and pulsed twice at 1,700 V and 25 μF with a 10-s interval in a Bio-Rad Gene Pulser XCell electroporation system (Bio-Rad, Australia). Cells were transferred to SDM containing 10% FBS and 100 units/ml penicillin/streptomycin. After 24 h, the medium was supplemented with selection drugs, including puromycin (Stemcell Technologies, Australia) at 10 μg/ml or Geneticin (G418; MP Biomedicals, Australia) at 50 μg/ml or both (hygromycin B was omitted at this stage). The selected cell lines were maintained at 27°C in RPMI medium supplemented with 10% FBS. Cultures were differentiated to axenic amastigotes in RPMI medium adjusted to pH 5.5 using 20% FBS at 35°C.

### Fluorescence microscopy.

To visualize mitochondria and glycosomes using fluorescence microscopy, live promastigotes and amastigotes were gently centrifuged (1,000 rpm, 5 min, room temperature) and resuspended in medium containing 20 nM MitotrackerRED CMXRos (Molecular Probes, Eugene, OR, USA) in RPMI medium lacking FBS for 5 min at room temperature. Cells were pelleted and resuspended in PBS containing 1 μg/ml Hoechst 33342 (Molecular Probes, Eugene, OR, USA) before being directly settled onto coverslips that were coated with poly-l-lysine (Sigma-Aldrich, Australia) and sealed with nail polish. Alternatively, parasites were fixed prior to imaging as follows. Cells were harvested by centrifugation, washed once in PBS, and then fixed in 4% paraformaldehyde–PBS (15 min at 0°C) before being washed three times in PBS and then settled onto poly-l-lysine-coated coverslips. Images were captured using a Leica SP8 confocal microscope (inverted microscope; Leica Microsystems, Australia) and 488-nm, 552-nm, and 638-nm-wavelength laser lines and HyD detectors for fluorescence and LAS X SP8 software (Leica microsystems, Australia). The different dyes were imaged using sequential frame collection, and controls were used to exclude bleed-through. Images were acquired using a 100×/1.4 oil lens objective. Images were handled using imageJ (Fiji) software (98). Colocalization analysis was performed. All images were deconvolved by the use of Huygens Professional version 19.04 (Scientific Volume Imaging, The Netherlands; http://svi.nl) and the in/near object algorithm, with signal-to-noise ratios of 20 and 40 iterations. Fiji Coloc2 was used to calculate the Pearson’s correlation coefficient and Manders’ thresholded coefficients tM1 and tM2 using Costes’ automatic threshold regression on the deconvolved images.

For *in vitro* infection assays, cultured macrophages (2 × 10^5^) were attached on 13-mm-diameter glass coverslips in a 24-well plate and infected with adapted axenic L. major promastigotes as described above. Cells were fixed with 4% (wt/vol) paraformaldehyde–PBS for 20 min. Cells were then washed with PBS, and nuclei were stained with DAPI (4′,6-diamidino-2-phenylindole; Merck, Darmstadt, Germany) for 10 min in the dark at room temperature. After staining, the cells were washed three times with PBS and the coverslips were mounted in Fluoromount (Sigma-Aldrich, Steinheim, Germany).

Cells were examined with a Zeiss Axio Observer Z1 microscope (Carl Zeiss, Jena, Germany). Images were acquired and analyzed with ZEN (Blue Edition; Carl Zeiss, Jena, Germany). Fluorescence was recorded aund an AxioCam MRm camera (Carl Zeiss, Jena, Germany) with ×63 magnification and oil immersion. Infected cells and the number of the intracellular parasites were counted using ImageJ (Version 1.47m).

For cytofluometric analyses, 250-μl volumes of the culture from green fluorescent protein (GFP)-expressing (81) bacteria cocultured with nonexpressing strains were transferred into a fluorescence-activated cell sorter (FACS) tube, centrifuged, and resuspended in 300 μl CellFix (BD Biosciences) for fixation. The samples were stored at 4°C until measurement.
