# Supplementary figures and images for: Leishmania Encodes a Bacterium-like 2,4-Dienoyl-Coenzyme A Reductase That Is Required for Fatty Acid β-Oxidation and Intracellular Parasite Survival
Source: mBio. 2020 Jun 2;11(3):e01057-20. doi: 10.1128/mBio.01057-20 (PMC7267886; doi:10.1128/mBio.01057-20)

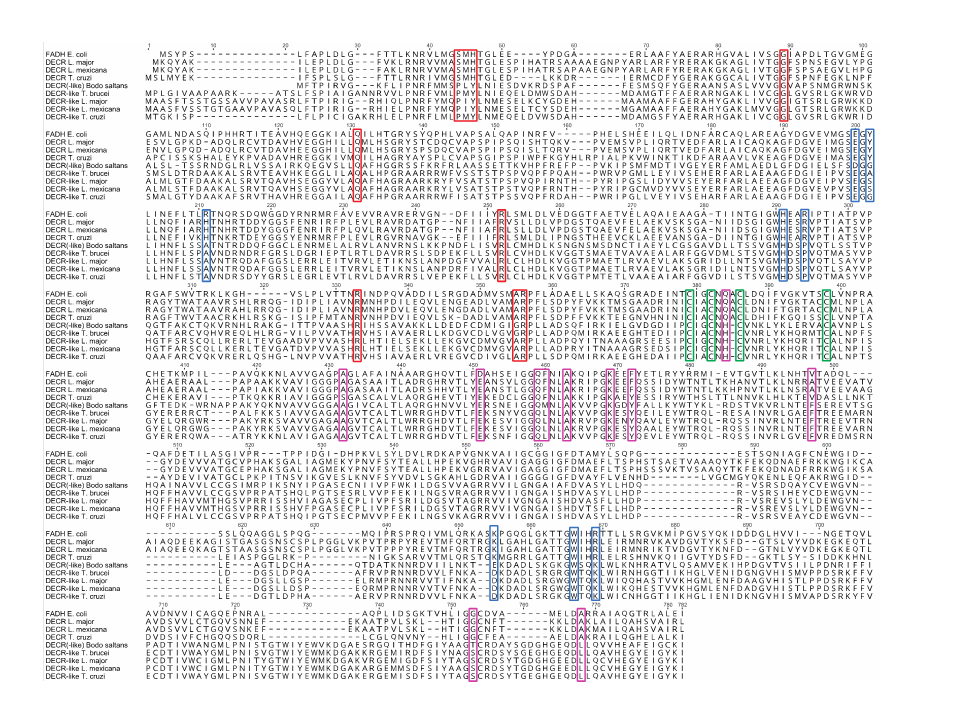

Supplement: FIG S2 [file mBio.01057-20-sf002.tif]

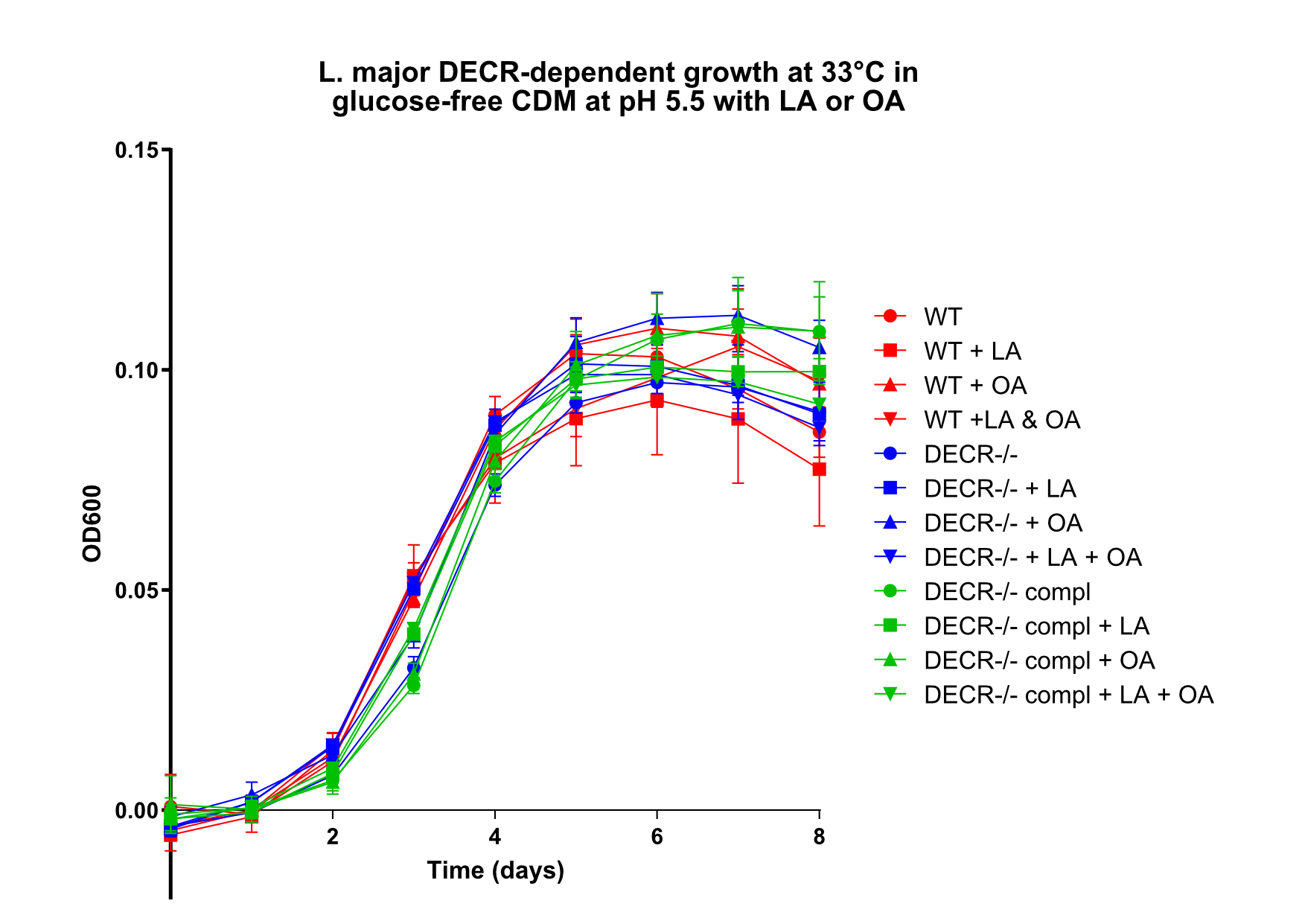

Supplement: FIG S3 [file mBio.01057-20-sf003.tif]

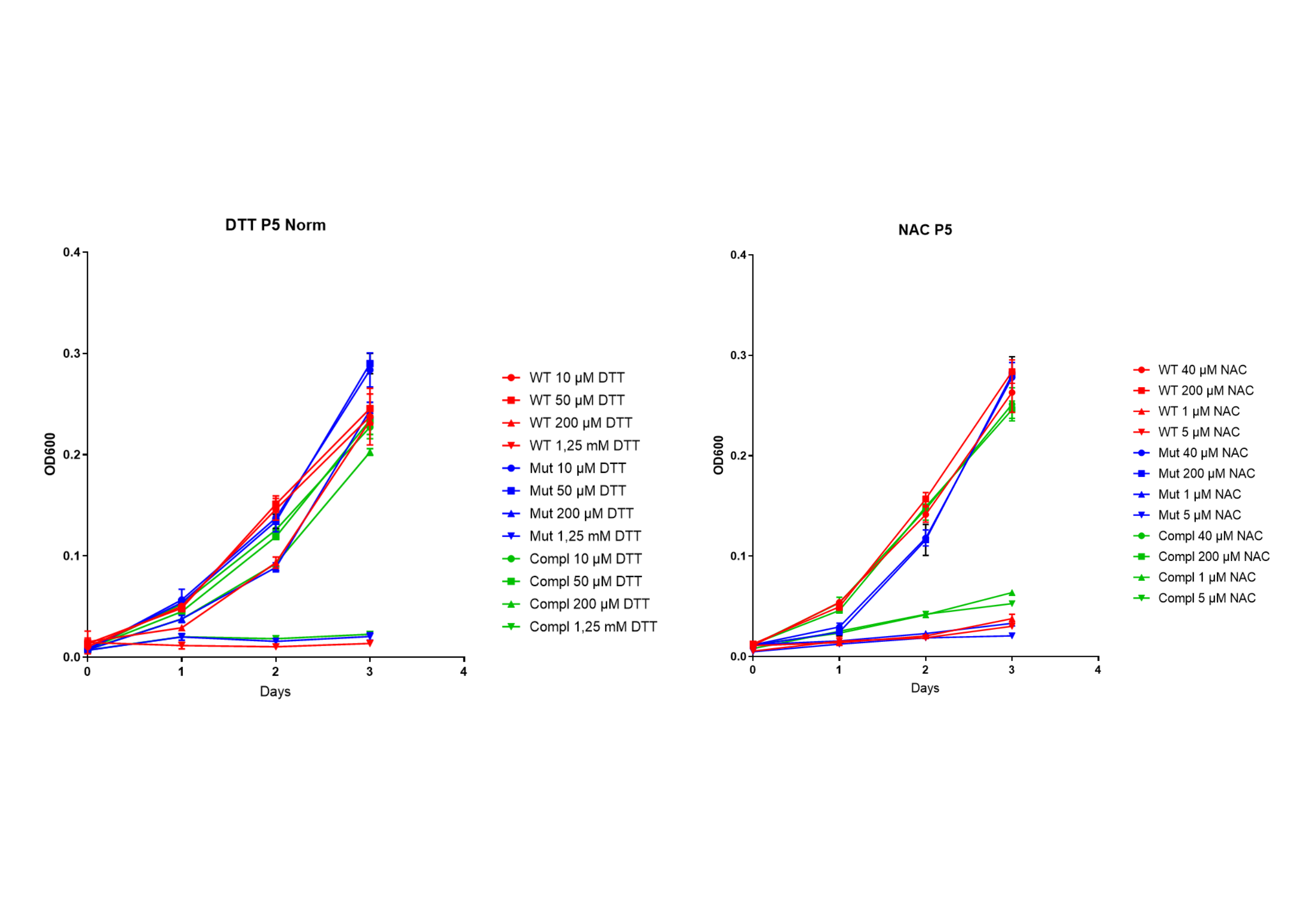

Supplement: FIG S4 [file mBio.01057-20-sf004.tif]
